# Active and productive ageing in India: evidence from the time use pattern of ageing adults

**DOI:** 10.1186/s12877-023-04428-6

**Published:** 2023-11-06

**Authors:** C. V. Irshad, P. Padma Sri Lekha, E. P. Abdul Azeez, S. Irudaya Rajan

**Affiliations:** 1 School of Social Sciences and Languages, Vellore Institute of Technology, Vellore, Tamil Nadu 632014 India; 2 The International Institute of Migration and Development, Thiruvananthapuram, Kerala 695011 India

**Keywords:** Active ageing, Productive ageing, Successful ageing, Older adults, India, J140, I140, C550, J250

## Abstract

**Background:**

With the increasing proportion of older adults in India, it becomes essential to get an insight into the various influencing factors of successful ageing. However, the literature on successful ageing is minuscule in the Indian context. The present study attempted to understand successful ageing in terms of active and productive ageing by exploring their determining factors.

**Methods:**

The data were extracted from the Longitudinal Ageing Study in India (LASI) Wave–1 (2017–2018). We utilized self-reported time use information from the experimental module of the LASI. A total of 7837 ageing adults were included in the study. We employed descriptive statistics, bivariate analysis and a multinominal logistic regression model to examine the prevalence and the determinants of active and productive ageing.

**Results:**

The prevalence of inactive ageing was higher among the Indian ageing population (57.47%), followed by active ageing (29.59%) and productive ageing (12.94%). Poor sleep quality and the prevalence of morbidity and disability limited the ageing population from attaining active and productive ageing. Engagement in physical activity was significantly associated with active and productive ageing (β = 0.83, 99% CI: -0.72–0.94 and β = 0.82, 99% CI: 0.66–0.98), respectively. Rural ageing adults were more likely to attain active ageing and less likely to attain productive ageing.

**Conclusion:**

Engagement in physical activities among the ageing population shall be promoted to attain active and productive ageing. Since the rural ageing population were less likely to attain productive ageing than their urban counterparts, opportunities to participate in more formal economic activities in rural areas could be promoted for the wellbeing of the second demographic dividend.

## Introduction

Ageing is an inevitable dynamic process in everyone’s life, but how individuals embrace it differs. The cumulation of cellular and molecular changes over the years slowly leads to physical and psychological decline, known as the biological ageing process [1]. Even though life span has dramatically increased in the past years [2], the decline of physical [3–6] and mental [7–9] strength during ageing has been inescapable. The proportion of ageing population across the globe has increased significantly, with the prediction that by 2050, low and middle-income countries will have a higher number of older people [1].

With increased empirical attention to ageing, various studies focus on how the ageing process can be made healthier [10]. Such attempts have resulted in different ageing ideals, including healthy, active, productive, and successful ageing [11]. These concepts may differ semantically, but they all direct to ‘ageing well’ [12]. Successful ageing has received attention recently, though the concept of positive ageing is traced back to the 1980s [13]. Successful ageing is a volatile concept, but the classic definition of the same by Rowe and Kahn was ‘high physical, social and psychological functioning in old age without major diseases’ [14]. Successful ageing is a multidimensional approach, with a biological perspective adapted to understand the psychological and social aspects of ageing [15]. A scoping review on how older adults perceive successful ageing identified twelve major themes, including positive thinking and attitudes, being healthy, financial security, acceptance and adaptation, engagement with life, spirituality, environment and social policy, social relationships and interaction, autonomy and independence, cognitive health, physical activity and having good death as the inevitable components [16].

Further, the ageing process leads to a decline in physical and mental abilities; however, its impact on overall functionality and wellbeing is minimal, leaving better scope for successful ageing [17]. On the other hand, active and productive ageing is about enhancing the opportunities for health, security, and participation to have a better quality of life while ageing [1, 18]. Active ageing is a broader term than productive ageing, which includes economic and non-economic activities [19], whereas productive ageing includes only economic activities [12]. It is clear from the above definitions that successful ageing encompasses active and productive ageing (Figure − 2).

Successful ageing is a dynamic and volatile process shaped by numerous aspects at community and individual levels, including physical activity. A study suggested that older adults involved in physical activity had a greater possibility of ageing successfully [20]. Further, recent studies indicate that an individual’s perception of ageing significantly influences the path of it [21–24]. In addition, a variety of psychosocial determinants, including perceived health status [25], leisure activity [26, 27], ego-integrity [28], economic status [29, 30], educational level [31, 32], social relationships and network [33, 34] influences the process of successful ageing.

Literature shows that the successful and active ageing process is complex and volatile with culture [35]. Having that said, it becomes essential to understand the dynamics of successful ageing in the Indian context. In addition, there is a pressing need to understand the nature of ageing in India, as the proportion of older adults is increasing globally [36]. It is also important to note that hardly any studies are conducted on successful ageing in the Indian setting. In this context, exploring successful ageing and the various factors determining it is significant. Hence, the present study attempted to understand successful ageing in terms of active and productive ageing in India through various factors such as health-related factors, socioeconomic and demographic variables, health risk behaviours and depression.

## Method

### Data and sample

The study utilized unit-level data from the first wave of the Longitudinal Ageing Study in India (LASI), conducted between April 2017 and December 2018. The survey investigated health, socioeconomic, and other relevant aspects of population ageing in India. The International Institute for Population Sciences (IIPS), Mumbai, designed and executed the survey with technical support from the University of Southern California (USC), the Harvard T H Chan School of Public Health (HSPH), and several other institutions [37]. The biennial panel survey adopted a three-stage sampling design in rural areas and a four-stage sampling design in urban areas.

**Fig. 1:**
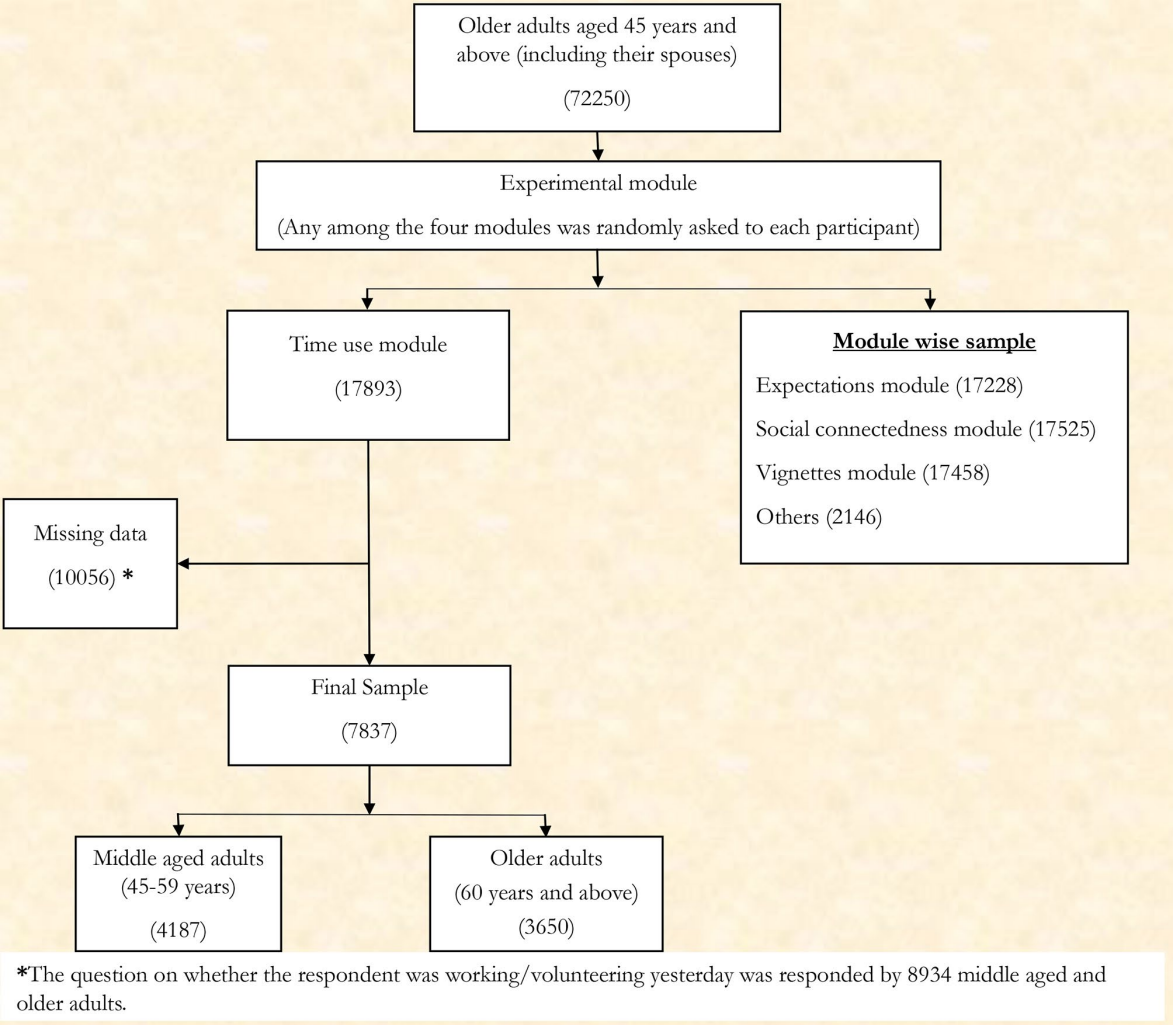
Sample selection procedure

The data were collected from selected and consented samples aged 45 years and above. The first wave of the LASI consisted of 72,250 older adults representing all Indian states and union territories (excluding Sikkim). The present study considered middle-aged and older adults aged 45 years and above. To study the active and productive ageing among the older adult population in India, we utilized the time-use section of the experimental module of the LASI survey. The experimental modules consist of four separate modules: (i) the Time Use module, (ii) the Expectations module, (iii) the Social connectedness module, and (iv) the Vignettes module. For every individual participant, any random module among these experimental modules was considered and utilized for data collection during the survey. The present study initially considered 17,893 individuals from the time-use module. Further, we considered cases only if the study participants responded to a question on their previous day’s work or volunteer activity (n = 8934). After excluding all missing cases, we had a final analytical sample of 7837 middle-aged and older adults. A detailed summary of the sample selection procedure is depicted in Fig. 1.

### Measures

#### Inactive, active and productive ageing

**Fig. 2:**
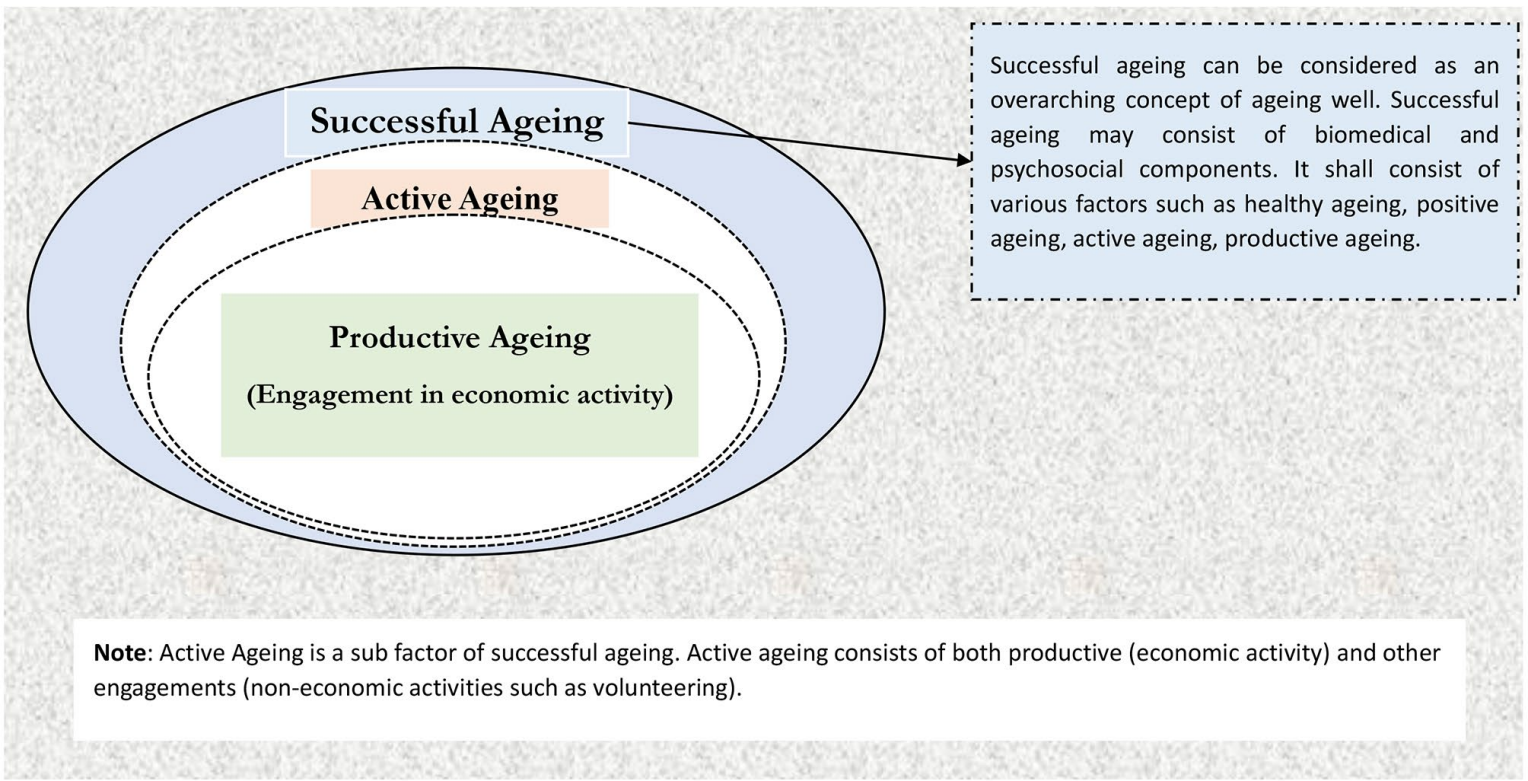
Conceptual framework of inactive ageing, active ageing and productive ageing

The concept of successful ageing is immensely relevant as the demographic transition has been taking place across the world. Successful ageing is yet to be conceptualized with more scientific rigour. A recent study aimed to conceptualize successful ageing pointed to the vagueness of the concept and identified that being active in different forms (both engagement in economic activities or otherwise) is considered one of the critical components of successful ageing [16]. Another study identified that successful ageing is considered a multidimensional concept embedded in biomedical and psychosocial factors, which can be measured using a mix of subjective and objective tools. The study also identified productive ageing as a subcomponent of successful ageing, whereas active ageing is a much broader concept than productive ageing [38]. Active ageing consists of both economic and non-economic activities [19]. Another study based on the active ageing policy from the European perspective showed that productive ageing is a narrow concept as it focuses on economic-related engagement, which is also gender-biased [12]. The present study aims to explore two of the subcomponents of successful ageing and their associated factors in the Indian context, i.e., (i) active ageing and (ii) productive ageing. Active and productive ageing offers more of a social aspect [11]. A summary of the conceptualization of the study is provided in Fig. 2.

##### Predictor variables

**Fig. 3:**
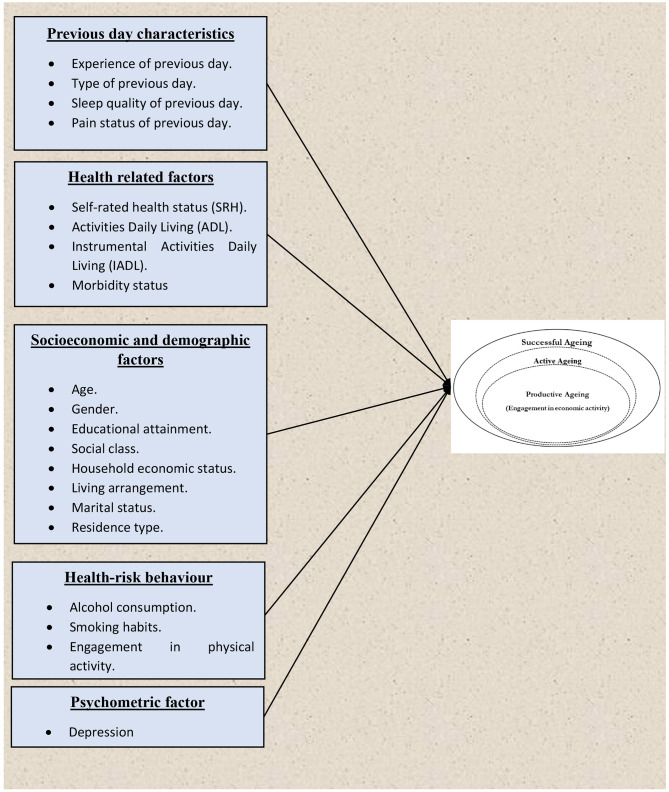
Summary of the conceptual framework of the study

In the present study, we have considered various predictor variables that influence active and productive ageing. This includes previous-day characteristics, health-related factors, health risk behaviours, psychometric factors, socioeconomic status, and demographics. A summary of the conceptual framework of the study is illustrated in Fig. 3.

##### Previous day characteristics

The study first considered the potential factors related to the previous day. We hypothesize that these factors could determine the active and productive ageing status. It is well established that the reliability of the recall of the most recent events is more [39]. Therefore, the previous day’s characteristic is a more reliable indicator, and four items were used to understand it in the study. First, this includes questions about the previous day’s experience (a normal day, an unusually stressful day, an unusually good day). Second, the type of previous day was understood based on whether it was a “weekday”, or “weekend”, or “holiday”. Third, the sleep quality of the previous day was categorized as “good” or “poor”. Fourth, the pain status of the previous day was categorized as “yes” or “no”.

##### Health-related factors

Previous studies have shown that health-related factors are unavoidable contributors to successful ageing [40]. This umbrella term consists of different health-related indicators, including self-rated health status (SRH), activities of daily living (ADL), instrumental activities of daily living (IADL) and morbidity status. The SRH was categorized as “good” (i.e., very good, good, and fair) and “poor” (poor and very poor). ADL and IADL were measured using questions on six and seven types of difficulties in everyday functioning, respectively. The responses for both ADL and IADL were classified as “high” (if no difficulty was reported) and “low” (if difficulty was reported in performing at least one of the items) [41, 42]. The morbidity status was measured based on nine diseases reported by the participants; the responses are categorized as “no disease”, “one disease”, and “multimorbidity”. The major diseases considered include hypertension, diabetes, cancer, chronic lung diseases, chronic heart diseases, stroke, arthritis, neurological or psychiatric issues and high cholesterol.

##### Socioeconomic Status and Demographics

 We considered a number of covariates based on socioeconomic and demographic factors. It consists of the age of the study population, which was divided into three categories: middle age (45–59 years), young older adults (60–69 years), and older adults (70 years and above). In addition, gender (male and female), educational attainment (no schooling, up to 5 years, 6–10 years, and above 10 years), social class (Scheduled Tribes (ST), Scheduled Castes (SC), Other Backward Class (OBC), and Others), household economic status (poorest, poorer, middle, richer and richest) were considered in the analysis. The study also considered additional variables such as living arrangement (living alone, with spouse and others, and with others), marital status (in a union and not in a union) and residence type (rural and urban). The expenditure data of each household on 29 non-food and 11 food items after standardizing the expenditure to a 30-day reference period was used to estimate household economic status [43].

##### Health-Risk Behaviour

It was established that health-risk behaviours are potential factors determining successful ageing [44, 45]. In this study, we have considered three health risk behaviour indicators such as ever consumption of alcohol (yes and no), ever smoker (yes and no), and engagement in vigorous physical activity (yes and no).

##### Psychometric factor

The literature has established a strong association between psychometric factors and late-life outcomes, including quality of life and successful ageing [46, 47]. In the present study, we considered depression status as a psychometric factor. LASI embraced the definition of depression as the experience of low mood and no pleasure in performing an activity that was earlier interesting for an extended period, i.e., at least for two weeks [48]. For the assessment of depression status, the Centre for Epidemiologic Studies Depression (CES-D) scale was used [49]. The short version of CES-D includes 10 -items (7 negatives and 3 positive feeling items). The composite score was calculated after reversing the score of 3 positive feelings items. The total CES-D score ranges between 1–10, with a higher score indicating depression symptoms. In the present study, based on the CES-D score, the individuals were classified into two groups, ‘yes’ with depressive symptoms if the score was 4 and above and ‘no’ indicating no depressive symptoms if the score was below 4 [50].

### Statistical analysis

We employed descriptive statistics and bivariate analysis to examine the preliminary results. In the multivariate model, we employed a multinomial logistic regression model. We opted for a multinomial logistic regression model since ordering outcome measures was difficult, though our outcome measure was a categorical variable [51]. We presented the results with estimated regression coefficients, and 90%, 95% and 99% confidence intervals are considered for drawing conclusions. The statistical analysis was performed using Stata Version 16.

## Results

The present study leaps to understand successful ageing through productive and active ageing in the Indian context using one of the world’s largest ageing databases, the LASI Wave 1. The present study included a total sample of 7837 older ageing adults, among which 4187 were middle-aged adults (45–59 years), and 3650 were older adults (60 years and above). Descriptive statistics, bivariate analysis and multinominal regression were employed to understand the prevalence of inactive, active and productive ageing and the determining factors.

**Fig. 4:**
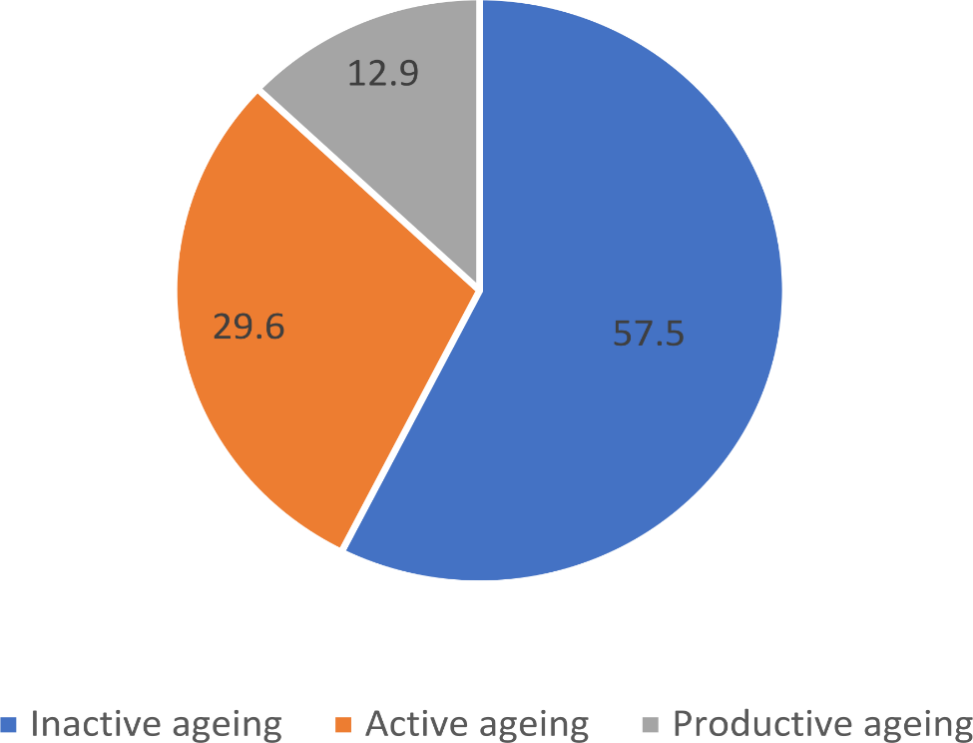
Prevalence of inactive, active, and productive ageing in India (%)

**Fig. 5:**
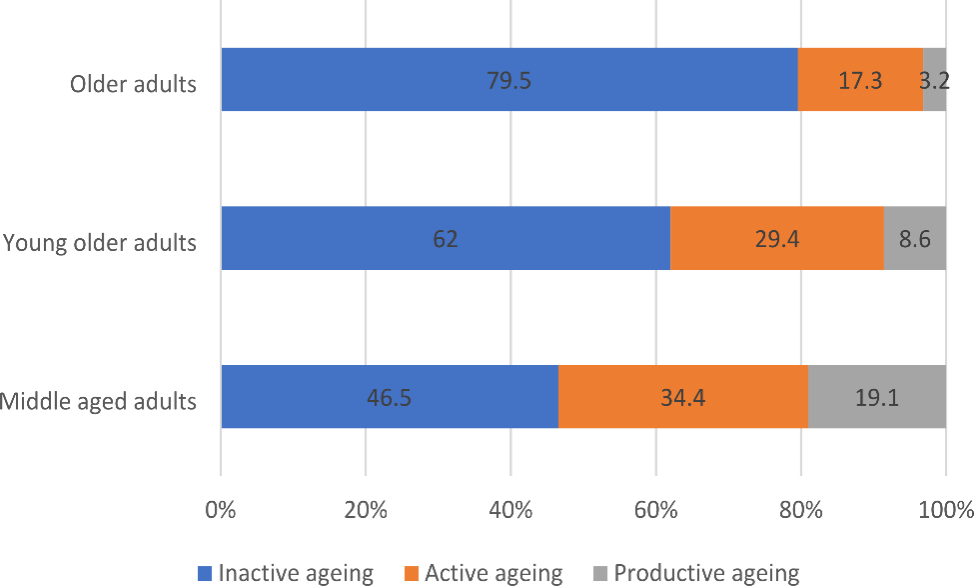
Prevalence of inactive, active, and productive ageing in India by age (%)

**Fig. 6:**
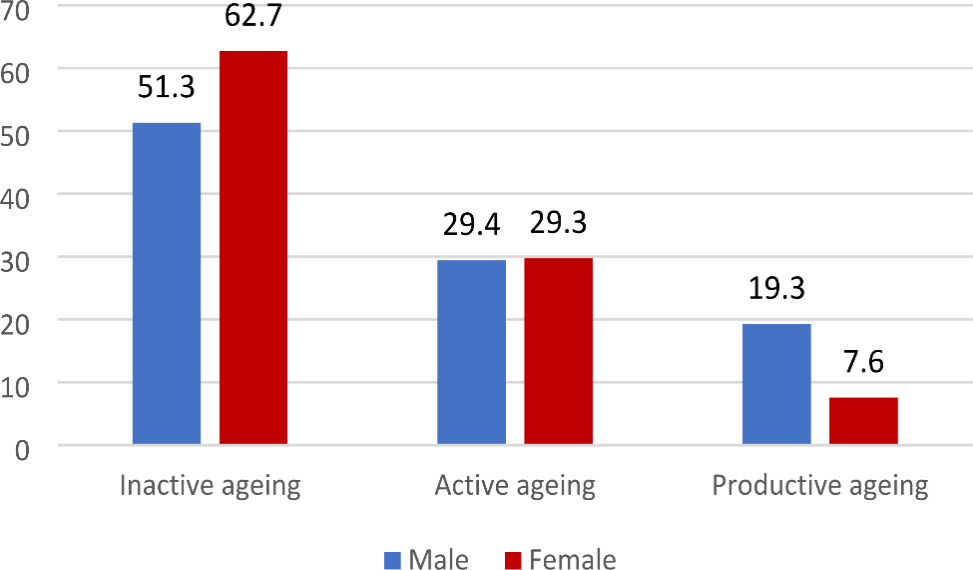
Prevalence of inactive, active, and productive ageing in India by gender (%)

It is evident from Fig. 4 that the prevalence of inactive ageing (57.5%) among ageing adults was higher when compared to ageing adults involved in active (29.6%) and productive ageing (12.9%). Figure 5 presents the prevalence of inactive, active and productive ageing by age groups. It was revealed that nearly 80% of the older adults aged 70 years and above were inactive. As expected, productive ageing was higher among middle-aged older adults (19.1%) compared to young older (8.6%) and older adults (3.2%). The preliminary results also indicated interesting observations on gender and successful ageing. Figure 6 shows that inactive ageing was higher among females than males (62.7% Vs 51.3%). On the contrary, productive ageing was higher among male ageing adults than female ageing adults (19.3% Vs 7.6%).

The descriptive analysis of the study sample is presented in Table 1. More than half of the study sample was middle-aged older adults (52.2%), followed by young old (27.6%) and oldest-old adults (20.2%). It was indicated that 93% of older ageing adults reported having a normal previous day. The adults who reported the previous day to be weekdays (72.4%) were more than those who reported weekends or holidays. A higher number of older adults reported a good quality of sleep (75.2%) and an unpainful experience on the previous day (93.3%). Based on health indicators, it was found that 15.7% and 36% of the study sample had low ADL and IADL, respectively. 17.8%, 27.3% and 17.6% of the study population reported poor SRH status, one disease and multimorbid conditions, respectively. It was noticed that more than half of the study sample were female ageing adults and had no formal schooling. It was found that only 3.2% of ageing adults were living alone, while nearly three-fourths of the ageing adults were living with their spouses and others. Just one-third of the study population were not in a marital union (25.4%) and urban residents (28.9%). Based on health risk behaviour, it was found that 14.8% and 38.1% of the study sample had ever consumed alcohol and were ever smokers, respectively and 42.6% of older adults engaged in vigorous physical activities. More than one-fourth of the ageing adults had depression symptoms.

Table 2 shows the prevalence of inactive, active, and productive ageing by background characteristics. It was found that inactive ageing was significantly higher among those who reported the previous day as a holiday. On the contrary, productive ageing was significantly high among those who reported the previous day as a weekday. It was observed that inactive ageing was significantly higher among those who reported poor sleep quality and painful experiences on the previous day. In contrast, their prevalence of active ageing was lower. It was identified that a significantly higher proportion of older adults who reported poor SRH had inactive ageing in contrast to those who reported good SRH status (72.8% Vs 54.1%: p < 0.01). Similarly, the prevalence of inactive ageing was significantly higher among those with low ADL and IADL compared to ageing adults with high ADL and IADL. Conversely, compared to those with poor SRH status, active and productive ageing prevalence was higher among ageing adults with good SRH status. It was also indicated that inactive ageing was significantly higher among ageing adults with morbid conditions, whereas active and productive ageing was higher among those with no morbid condition.

**Table 1:** Descriptive characteristics of study variables

Variable	Sample distribution	
	**Frequency**	**W%**
Experience of the previous day		
Normal	7364	93
Unusually stressful	234	3
Unusually good day	239	4
Type of last day		
Weekday	5686	72.4
Weekend	1101	14.2
Holiday	1050	13.4
Sleep quality of last day		
Good	5917	75.2
Poor	1920	24.8
Was yesterday painful		
No	7358	93.3
Yes	479	6.7
Self-rated health (SRH)		
Good	6532	82.2
Poor	1305	17.8
Activities of Daily Living (ADL)		
High	6736	84.3
Low	1101	15.7
Instrumental Activities of Daily Living (IADL)		
High	5258	64
Low	2579	36
Morbidity		
None	4207	55.1
One disease	2115	27.3
Multimorbidity	1515	17.6
Age		
45–59 years	4187	52.2
60–69 years	2222	27.6
70 and above years	1428	20.2
Gender		
Female	4224	54
Male	3613	46
Education		
No schooling	3673	50.5
Up to 5 years	1477	19
6–10 years	1877	20.2
More than 10 years	810	10.3
Social class		
Scheduled Tribes (ST)	1281	8.8
Scheduled Castes (SC)	1336	19.3
Other Backward Class (OBC)	3056	46.2
Others	2164	25.7
Household wealth background		
Poorest	1581	21.9
Poorer	1567	20.9
Middle	1619	21.9
Richer	1561	18.6
Richest	1509	16.7
Living arrangement		
Alone	255	3.2
With spouse and others	5755	73.3
With others	1827	23.5
Marital status		
In a union	5908	74.6
Not in a union	1929	25.4
Residence type		
Urban	2691	28.9
Rural	5146	71.1
Ever consume alcohol		
No	6419	85.2
Yes	1418	14.8
Ever smoke		
No	4951	61.9
Yes	2886	38.1
Physical activity		
No	4683	57.4
Yes	3154	42.6
Depression		
No	5854	72.4
Yes	1983	27.6
Total	**7837**	**100**

Table 2 further indicated that inactive ageing increased with age while active and productive ageing declined and was statistically significant. It was found that females had a significantly higher prevalence of inactive ageing than males (62.7% Vs 51.3%: p < 0.01). Concerning productive ageing, it was found that male ageing adults had a significantly higher prevalence than female ageing adults (19.3% Vs 7.6%: p < 0.01). Interestingly, the results indicated that active ageing was significantly higher among rural ageing adults in comparison to their urban counterparts (32.5% Vs 22.5%: p < 0.01), whereas productive ageing was higher among urban ageing adults in comparison to their rural counterparts (16.2% Vs 11.6%: p < 0.01). The results showed that inactive ageing was significantly higher among adults who did not engage in physical activity. In comparison, active and productive ageing was significantly higher among those who engaged in physical activities.

**Table 2:** Prevalence of inactive, active, and productive ageing by background characteristics

Variables	Prevalence of inactive, active, and productive ageing (W%)					
	Inactive ageing	P value	Active ageing	P Value	Productive ageing	P value
Experience of previous day						
Normal	57.1	0.55	29.4	0.93	13.5	0.12
Unusually stressful	64.9		28.2		6.9	
Unusually good day	59.7		35.5		4.8	
Type of last day						
Weekday	56.0	< 0.01	29.8	< 0.01	14.2	< 0.01
Weekend	55.4		33.5		11.1	
Holiday	67.5		24.3		8.2	
Sleep quality of last day						
Good	56.3	< 0.01	30.8	< 0.01	12.9	< 0.05
Poor	61		25.9		13.1	
Was yesterday painful						
No	56.7	< 0.01	30.1	< 0.05	13.2	< 0.05
Yes	68.3		22.6		9.1	
Self-rated health (SRH)						
Good	54.1	< 0.01	31.7	< 0.01	14.2	< 0.01
Poor	72.8		19.8		7.4	
Activities of Daily Living (ADL)						
High	54.8	< 0.01	31.5	< 0.01	13.7	< 0.01
Low	71.7		19.5		8.8	
Instrumental Activities of Daily Living (IADL)						
High	52.1	< 0.01	31.9	< 0.01	16	< 0.01
Low	67.1		25.3		7.6	
Morbidity						
None	52	< 0.01	32	< 0.01	16	< 0.01
One disease	60.3		28.9		10.8	
Multimorbidity	70.3		23.2		6.5	
Age						
45–59 years	46.5	< 0.01	34.4	< 0.01	19.1	< 0.01
60–69 years	62		29.4		8.6	
70 and above years	79.5		17.3		3.2	
Gender						
Female	62.7	< 0.01	29.7	0.81	7.6	< 0.01
Male	51.3		29.4		19.3	
Education						
No schooling	58.3	0.35	30.2	< 0.01	11.5	< 0.01
Up to 5 years	58.9		26.5		14.6	
6–10 years	58.4		28		13.6	
More than 10 years	48.9		35.3		15.8	
Social class						
Scheduled Tribes (ST)	49.4	< 0.01	38.1	< 0.01	12.5	< 0.01
Scheduled Castes (SC)	58.9		25.7		15.4	
Other Backward Class (OBC)	55.2		31.8		13	
Others	63.1		25.6		11.3	
Household economic background						
Poorest	57.7	< 0.05	32.3	0.7	10	0.09
Poorer	55.9		30.4		13.7	
Middle	58.5		26		15.5	
Richer	54.5		31.2		14.3	
Richest	61.1		27.8		11.1	
Living arrangement						
Alone	63.1	< 0.01	20.1	< 0.01	16.8	< 0.01
With spouse and others	53.9		32.1		14	
With others	67.9		22.9		9.2	
Marital status						
In a union	54.1	< 0.01	32	< 0.01	13.9	< 0.01
Not in a union	67.5		22.5		10	
Residence type						
Urban	61.3	< 0.01	22.5	< 0.01	16.2	< 0.01
Rural	55.9		32.5		11.6	
Ever consume alcohol						
No	58.6	< 0.01	29.1	< 0.01	12.3	< 0.01
Yes	51.1		32.6		16.3	
Ever smoke						
No	59.9	< 0.01	28.3	< 0.01	11.8	< 0.01
Yes	53.4		31.8		14.8	
Physical activity engagement						
No	70.5	< 0.01	21.2	< 0.01	8.3	< 0.01
Yes	39.9		40.8		19.3	
Depression						
No	57.8	0.11	29.8	0.28	12.4	0.36
Yes	56.5		29		14.5	
Total	**57.5**		**29.6**		**12.9**	

The results of the multinomial logistic regression analysis are presented in Table 3. It was revealed that those ageing adults who reported the previous day as a weekend were less likely to have productive ageing relative to the likelihood of inactive ageing (β=-0.23, 95% CI: -0.46 - -0.01). Further, individuals who reported the previous day as a holiday were less likely to have active (β=-0.26, 99% CI: -0.42 - -0.10) and productive ageing (β=-0.49, 99% CI: -0.74 - -0.24) relative to the likelihood of inactive ageing. It was revealed that those who reported poor sleep quality on the previous day were less likely to have active (β=-0.21, 99% CI: -0.33 - -0.08) and productive ageing (β=-0.18, 90% CI: -0.37 - -0.01) relative to the likelihood of inactive ageing.

Based on health-related indicators, it was found that ageing adults with poor self-rated health were also less likely to experience active ageing compared to individuals with good self-rated health (β=-0.26, 99% CI: -0.42 - -0.09). Similarly, older adults with low ADL were less likely to experience active (β=-0.19, 95% CI: -0.37 - -0.02) and productive ageing (β=-0.28, 95% CI: -0.57 - -0.01) compared to individuals with high ADL status. In the case of IADL status, compared to ageing adults with high IADL, those with low IADL status were less likely to experience active ageing relative to the likelihood of inactive ageing (β=-0.13, 90% CI: -0.26 - -0.00). Individuals with multimorbidity conditions had a lower inclination towards active and productive ageing relative to the higher risk of falling into inactive ageing compared to adults with no diseases.

**Table 3:** Multinomial Logistic regression of active and productive ageing by background characteristics

Variables	Active ageing (CI)	Productive ageing (CI)
**Experience of previous day (Ref: Normal)**		
Unusually stressful	0.05 (-0.27–0.36)	0.01 (-0.43–0.46)
Unusually good days	-0.15 (-0.45–0.16)	-0.69* (-1.21 - -0.17)
**Type of last day (Ref: Weekday)**		
Weekend	0.01 (-0.14–0.16)	-0.23^$^ (-0.46 - -0.01)
Holiday	-0.26* (-0.42 - -0.10)	-0.49* (-0.74 - -0.24)
**Sleep quality of last day (Ref: Good)**		
Poor	-0.21* (-0.33 - -0.08)	-0.18^#^ (-0.37–0.00)
**Was yesterday painful (Ref: No)**		
Yes	-0.11 (-0.35–0.13)	-0.16 (-0.53–0.20)
**Self-rated health (SRH) (Ref: Good)**		
Poor	-0.26* (-0.42 - -0.09)	-0.17 (-0.42–0.08)
**Activities of Daily Living (ADL) (Ref: High)**		
Low	-0.19^$^ (-0.37 - -0.02)	-0.28^#^ (-0.57–0.01)
**Instrumental Activities of Daily Living (IADL) (Ref: High)**		
Low	-0.13^#^ (-0.26–0.00)	-0.16 (-0.36–0.04)
**Morbidity (Ref: None)**		
One disease	-0.01 (-0.14–0.12)	-0.16^#^ (-0.34–0.03)
Multimorbidity	-0.17^$^ (-0.33 - -0.01)	-0.43* (-0.67 - -0.20)
**Age category (Ref: 45–59 years)**		
60–69 years	-0.26* (-0.39 - -0.14)	-0.93* (-1.12 - -0.75)
70 years and above	-0.76* (-0.94 - -0.59)	-2.22* (-2.58 - -1.87)
**Gender (Ref: Female)**		
Male	-0.05 (-0.19–0.08)	1.05* (0.86–1.25)
**Education (Ref: No schooling)**		
Up to 5 years	-0.18^$^ (-0.33 - -0.03)	-0.13 (-0.35–0.09)
6–10 years	-0.14^#^ (-0.29–0.01)	-0.17 (-0.37–0.04)
More than 10 years	-0.05 (-0.26–0.16)	-0.02 (-0.29–0.26)
**Social class (Ref: Scheduled Tribe (ST)**		
Scheduled Castes (SC)	-0.21^$^ (-0.40 - -0.02)	0.56* (0.29–0.83)
Other Backward Class (OBC)	0.17^$^ (0.02–0.33)	0.54* (0.30–0.78)
Others	-0.06 (-0.24–0.11)	0.26^#^ (-0.01–0.53)
**Household economic background (Ref: Poorest)**		
Poorer	0.03 (-0.14–0.19)	0.08 (-0.16–0.33)
Middle	-0.03 (-0.20–0.14)	0.34* (0.11–0.58)
Richer	-0.02 (-0.19–0.15)	0.13 (-0.11–0.38)
Richest	-0.13 (-0.31–0.05)	0.02 (-0.24–0.28)
**Living arrangement (Ref: Alone)**		
With spouse and others	-0.05 (-0.55–0.45)	-0.93* (-1.54 - -0.32)
With others	0.01 (-0.34–0.36)	-0.83* -1.26 - -0.41)
**Marital Status (Ref: In a union)**		
Not in a union	-0.33 (-0.73–0.08)	0.10 (-0.43–0.62)
**Residence type (Ref: Urban)**		
Rural	0.44* (0.31–0.57)	-0.32* (-0.49 - -0.14)
**Ever consume alcohol (Ref: No)**		
Yes	0.03 (-0.13–0.18)	-0.09 (-0.29–0.11)
**Ever smoke (Ref: No)**		
Yes	0.13^$^ (0.01–0.26)	0.14 (-0.04–0.31)
**Physical activity engagement (Ref: None)**		
Yes	0.83* (0.72–0.94)	0.82* (0.66–0.98)
**Depression (Ref: No)**		
Yes	0.10 (-0.03–0.23)	0.15 (-0.03–0.33)
Constant	-0.82* (-1.37 - -0.28)	-1.15* (-1.85 - -0.46)
**Sample**	**7,837**	**7,837**

CI: Confidence Interval in parentheses: * p < 0.01, ^$^ p < 0.05, ^#^ p < 0.1

Among the factors related to socioeconomic and demographic domains, the results revealed that young older adults and older adults were less likely to attain active ageing relative to inactive ageing in comparison to middle-aged adults (β=-0.26, 99% CI: -0.39 - -0.14, and β=-0.76, 99% CI: -0.94 - -0.59, respectively). Similarly, it was also disclosed that young older adults and older adults were less likely to attain productive ageing relative to inactive ageing than middle-aged adults (β=-0.93, 99% CI: -1.12 - -0.75 and β=-2.22, 99% CI: -2.58 - -1.87, respectively). The evidence indicated a significant gender difference in productive ageing. It was shown that male ageing adults were more likely to attain productive ageing relative to the risk of inactive ageing than female ageing adults (β=-1.05, 99% CI: 0.86–1.25). Social class variable indicated that compared to older adults from ST background, ageing adults from SC, OBC and Other social classes were more likely to attain productive ageing relative to the risk of inactive ageing. Household economic status and educational attainment did not reveal a consistent effect on active and productive ageing. However, it was reported that in comparison with ageing adults with no formal schooling, those who earned up to five years and 6–10 years of education were less likely to experience active ageing relative to the risk of falling into inactive ageing (β= -0.18, 95% CI: -0.33–0.03 and β= -0.14, 90% CI: -0.29–0.01, respectively).

A notable result from the study is that compared to older adults who lived alone, those who lived with a spouse and others (β= -0.93, 99% CI: -1.54 – -0.32) and with others (β= -0.83, 99% CI: -1.26 – -0.41) were less likely to attain productive ageing. The results indicated that rural ageing adults were more likely to experience active ageing (β = 0.44, 99% CI: 0.31–0.57) and less likely to attain productive ageing (β= -0.32, 99% CI: -0.49–0.14) in comparison to their urban counterparts. Interestingly, the study results disclosed that in comparison to ageing adults who did not engage in physical activities, those engaged in physical activities were more likely to attain both active and productive ageing relative to a risk of inactive ageing (β = 0.83, 99% CI: 0.72–0.94 and β = 0.82, 99% CI: 0.66–0.98, respectively).

## Discussion

In this study, we attempted to generate insights into the various influential aspects of a successful ageing phenomenon. The results of this study indicated that more than 50% of the sample experienced inactive ageing. Similarly, a study with LASI data supported this result by reporting that about 31% and 59% of older adults did not engage in moderate to vigorous physical activity [52]. Further, inactivity leads to irreversible changes due to ageing [53]. Engaging in physical activity and being physically active is vital for older adults to attain active and productive ageing. Our result also pointed out that inactive ageing among older adults increases with age, which is consistent with previous studies [54, 55]. Further, the study indicated that there was a significant gender difference in terms of inactive and productive ageing. In contrast, a multi-country study using an individual-level index of active ageing indicated a mixed evidence of gender difference [56]. Generally, males are more likely to experience productive ageing, as indicated by the present study results, which could be attributed to lower levels of economic participation of the ageing female population in developing countries like India. Further, the variations in gender could be attributed to cultural underpinnings as the economic aspects are culturally associated with males over females. Therefore, improving the requisites for productive ageing of female older adults should be a policy priority.

The results showed that older adults with poor sleep quality on the previous day had a high risk of inactive ageing relative to the likelihood of active and productive ageing. An earlier study suggested that sleep quality is an important indicator of health, wellbeing, and adaptation, which could potentially determine the facets of successful ageing [57]. In addition, a bidirectional association exists between pain and sleep quality [58], which may contribute to the ageing process. This study results showed that those who experienced pain on the previous day were at high risk of inactive ageing, which is consistent with previous study findings [59, 60]. Literature established that pain and inactivity have a bidirectional relationship [61], where the fear component makes older adults with chronic pain more vulnerable to inactivity [62].

Notably, individuals who reported good self-rated health and no morbid condition were likelier to attain active and productive ageing relative to the risk of inactive ageing. Similar to the present study results, a study indicated that self-rated health was associated with active and healthy ageing among older adults [15]. Similarly, studies indicated multimorbidity was common among inactive older adults [63–65]. Further, a longitudinal study of 10 years based on 10 chronic conditions conducted in Taiwan established that prolonged morbid years hindered successful ageing [66]. Based on disability status, older adults with lower ADL and IADL status were less likely to be active and productive. Disability is one of the obstacles to independent physical and social functioning among older adults, which may lead to inactivity. A study disclosed that increased difficulties in daily activity were associated with a decline in cognitive abilities [67]. As defined in the conceptual framework of successful ageing, cognition is an important component which could tie in with active and productive ageing (Refer to Fig. 2). Since successful ageing is a broader concept that encapsulates active, productive and cognitive ageing, their interrelation needs to be explored further [68]. Generally, it is established that the social and physical skills of those who face difficulties in basic functioning are at high risk, which may limit attaining successful ageing [69, 70].

Active ageing was significantly influenced by educational level. The evidence indicated that compared to those with no schooling, those with some years of education were less likely to experience active ageing. Our result contradicts expectations as studies have indicated (both formal and non-formal education) was a remarkable part of successful ageing [71]. Further, the present result may be because life-long learning promotes newer skills during middle and late adulthood, paving the way for productive ageing [72, 73]. Results showed that compared to older adults from the poorest economic backgrounds, adults from higher economic backgrounds were more likely to achieve healthy ageing. However, the result was significant only for middle-class economic status, consistent with a previous study [29]. Regarding living arrangements, individuals who lived alone had a greater inclination towards productive ageing than ageing adults who lived with spouses and others or only with others. However, evidence from the literature is diverse, as a study suggested that married older adults living only with a spouse had higher chances of successfully ageing than individuals living with others [74]. Another recent study indicated a significant association between living alone and successful ageing [75]. A higher inclination of productive ageing among those older adults living alone is possible as they may be engaged in productive activities for survival.

The study results highlighted that those ageing adults in rural areas had a higher likelihood of active ageing. In contrast, ageing adults who resided in urban areas had a higher likelihood of productive ageing. These results were concurrent with earlier studies [76, 77]. In urban areas, productivity chances would be higher as people find more economic activities and job opportunities than in rural areas, leading to productive ageing. Similarly, in rural areas, people are expected to engage in unpaid activities, which could lead to active ageing in rural areas [78].

It is essential to note that involvement in physical activity has a significant effect on successful ageing. A systematic review suggested that older adults aged 60 years and above who were physically active had a lower risk of various physical and mental health complications along the trajectories of ageing well [79]. Depression was not a significant predictor of successful ageing in the present study, possibly due to the lower percentage of older adults with depression in this sample. In contrast, earlier studies showed that depression is a major barrier to successful ageing [46, 80, 81].

The study’s main strengths include using large-scale data from the study sample. Further, the study offers an earlier piece of evidence of active and productive ageing in the Indian context. However, the study is not free from potential limitations. Firstly, the study used a cross-sectional approach, which limits the establishment of cause-and-effect relationships among the study variables. The results in the present study only indicate the association among study variables. This could be addressed in the future as LASI is an ongoing longitudinal study. Secondly, the study used many self-rated information by older adults as key variables, including self-rated health status, self-reported morbidity status etc. Even though such self-rated health information can be used as reliable indicators [82], older adults may be misreporting as they are generally at high risk of reporting bias [83]. Finally, the present study is limited to active and productive ageing, interconnected components of successful ageing. Future studies may explore other dimensions of successful ageing and their interconnectedness to understand ageing better.

## Conclusion

The study presents one of the earlier pieces of evidence on the prevalence of inactive ageing and various factors that promote active and productive ageing in the Indian context. Ageing is unavoidable, but making it healthier and more successful is a public health priority. This study paves the way for developing future interventions as there is a pressing need for addressing the complexities of ageing, especially in a rapidly ageing context in India. The study points to the importance of sleep quality in determining active and productive ageing. The results further showed that the prevalence of morbidity and disability limited the ageing population from attaining active and productive ageing. Engagement in physical activities among the ageing population shall be promoted to attain active and productive ageing. Since the rural ageing population were less likely to attain productive ageing than their urban counterparts, opportunities to participate in more formal economic activities in rural areas could be promoted for the wellbeing of the ageing population, the second demographic dividend.

## Data Availability

The data used for this study is available through the following website. https://www.iipsindia.ac.in/content/lasi-wave-i or through https://g2aging.org/.
